# Supplementary material for: RNA G-quadruplexes control mitochondria-localized mRNA translation and energy metabolism
Source: Nat Commun. 2025 Apr 7;16:3292. doi: 10.1038/s41467-025-58118-5 (PMC11977240; doi:10.1038/s41467-025-58118-5)
Supplement: Supplementary file 1 — Supplementary Information [file 41467_2025_58118_MOESM1_ESM.pdf]

a

RNA G-quadruplexes		GO term	Annotated	Significant	pv.fisher.BH
Experimentally identified<br><br>RG4-seq, *in vitro*	*Kwok_2016* (K+; PDS)	Mitochondrion	1570	550	2.16E-15
		Mitochondrial part	1023	344	1.96E-07
		Mitochondrial outer membrane	180	74	6.44E-05
		Mitochondrial matrix	462	161	1.04E-04
	Guo_2016 (K+)	Mitochondrion	1570	463	2.04E-09
		Mitochondrial outer membrane	180	67	8.19E-05
Predicted<br><br>Algorithm, GQ score > 21		Mitochondrion	1570	1090	6.64E-06
		Mitochondrial outer membrane	180	140	2.65E-04

b

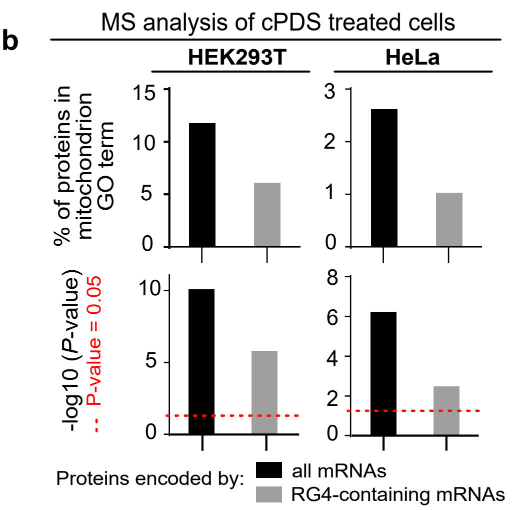

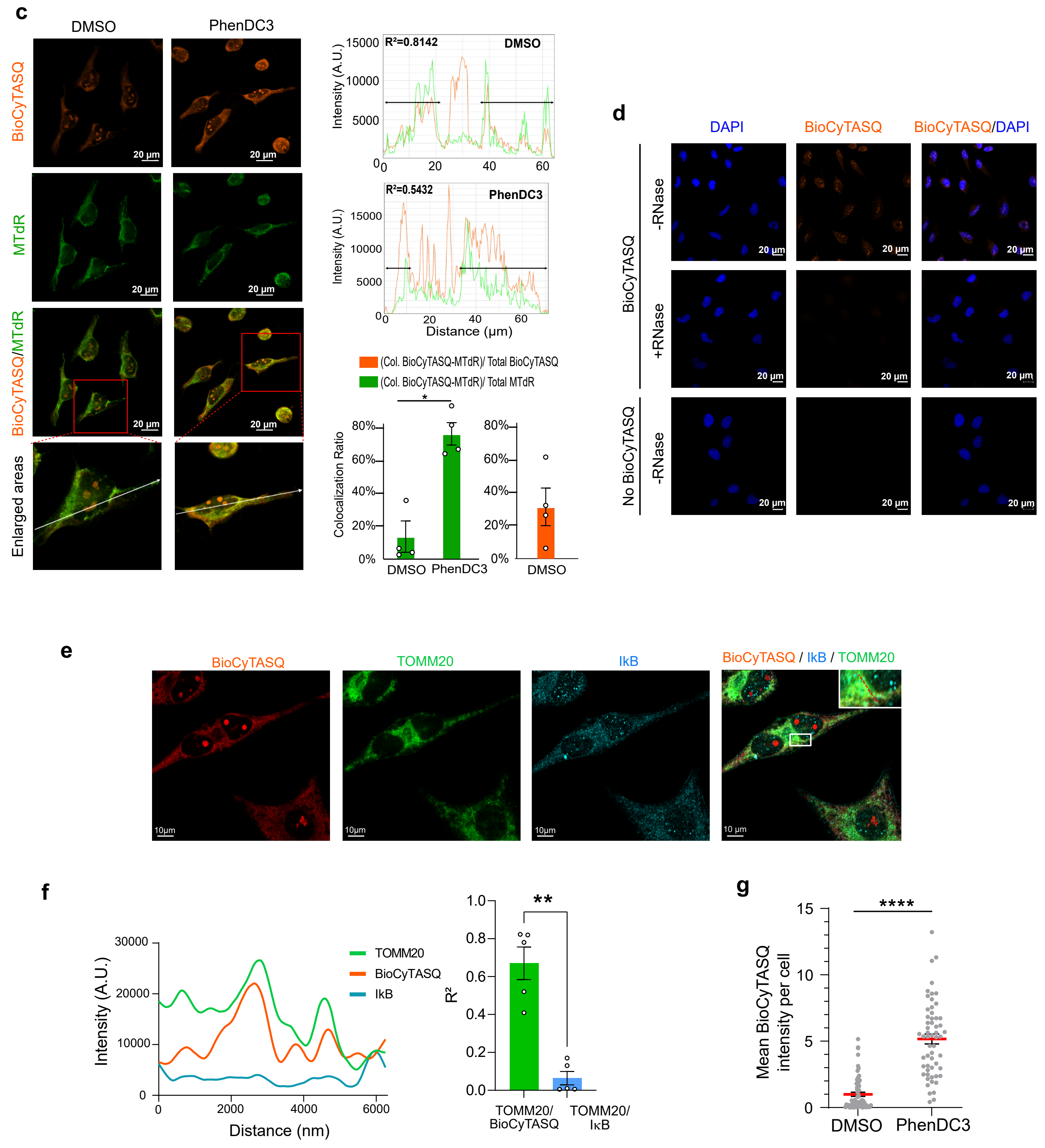

Supplementary Figure 1 (related to Figure 1). RG4 localization in mitochondrial mRNAs (a) Gene ontology enrichment analysis of RG4-containing mRNAs either experimentally validated based on [1,2] or predicted based on [3]. **(b)** Gene ontology enrichment analysis, along with the -log10 *P*-value of the enrichment, of proteins encoded by mRNAs containing predicted RG4s and identified by mass spectrometry (MS) analysis to be differentially expressed after 20 µM carboxypyridostatin (cPDS) treatment for 48 h in HEK293T or HeLa cells (n=5 independent experiments). **(c)** RG4 detection in live cells using the BioCyTASQ probe (1 µM for 24 h) and mitochondria labelling using MitoTracker Deep Red (MTdR) of HeLa cells treated with DMSO or 20 µM of PhenDC3 for 24 h. Graphs display the fluorescence intensity (arbitrary unit) in each channel over the distance depicted by the white arrows. The correlation coefficient (R^2) between the fluorescence intensities in the cytoplasm (depicted by the black arrows) is indicated. Scale bar indicates 20 µm. Shown is a representative result from n=4 independent experiments. The histograms display the the colocalization between the BioCyTASQ and MTdR signals using the ratio of colocalized areas over total MTdR area (green bars) or the ratio of colocalized areas over total BioCyTASQ area (orange bar). Data are presented as mean values ± SEM of n=4 independent experiments, *P*-value= 0.0253 (two-sided paired t-test). **(d)** Labeling of HeLa cells with or without BioCyTASQ (treatment of live cells with 1 µM BioCyTASQ for 24 h), with DAPI and treated or not with RNase A/T1. Shown is a single representative field from one experiment over n=3 independent experiments. Scale bar indicates 20 µm. **(e)** RG4 detection in fixed cells using the BioCyTASQ probe (1 µM for 24 h) coupled with an immunofluorescence analysis of TOMM20 and IkB protein localization in HeLa cells. Scale bar indicates 10 µm. **(f)** Graphs displaying the fluorescence intensity (arbitrary unit) in each channel over the distance depicted by the red arrow in (e). The correlation coefficient (R^2) of the colocalized fluorescence intensities of BioCyTASQ and IkB with TOMM20 signal is indicated and calculated in 5 different cells, *P*-value= 0.001 (two-sided paired t-test). Source data are provided as a Source Data file. **(g)** Quantification of the BioCyTASQ signal intensity from Supplementary Fig. 1c plotted relatively to the DMSO condition. Data are presented as mean values ± SEM of n=4 independent experiments and each dot is a cell, *P*-value= 4,695e-16 (two-sided paired t-test).

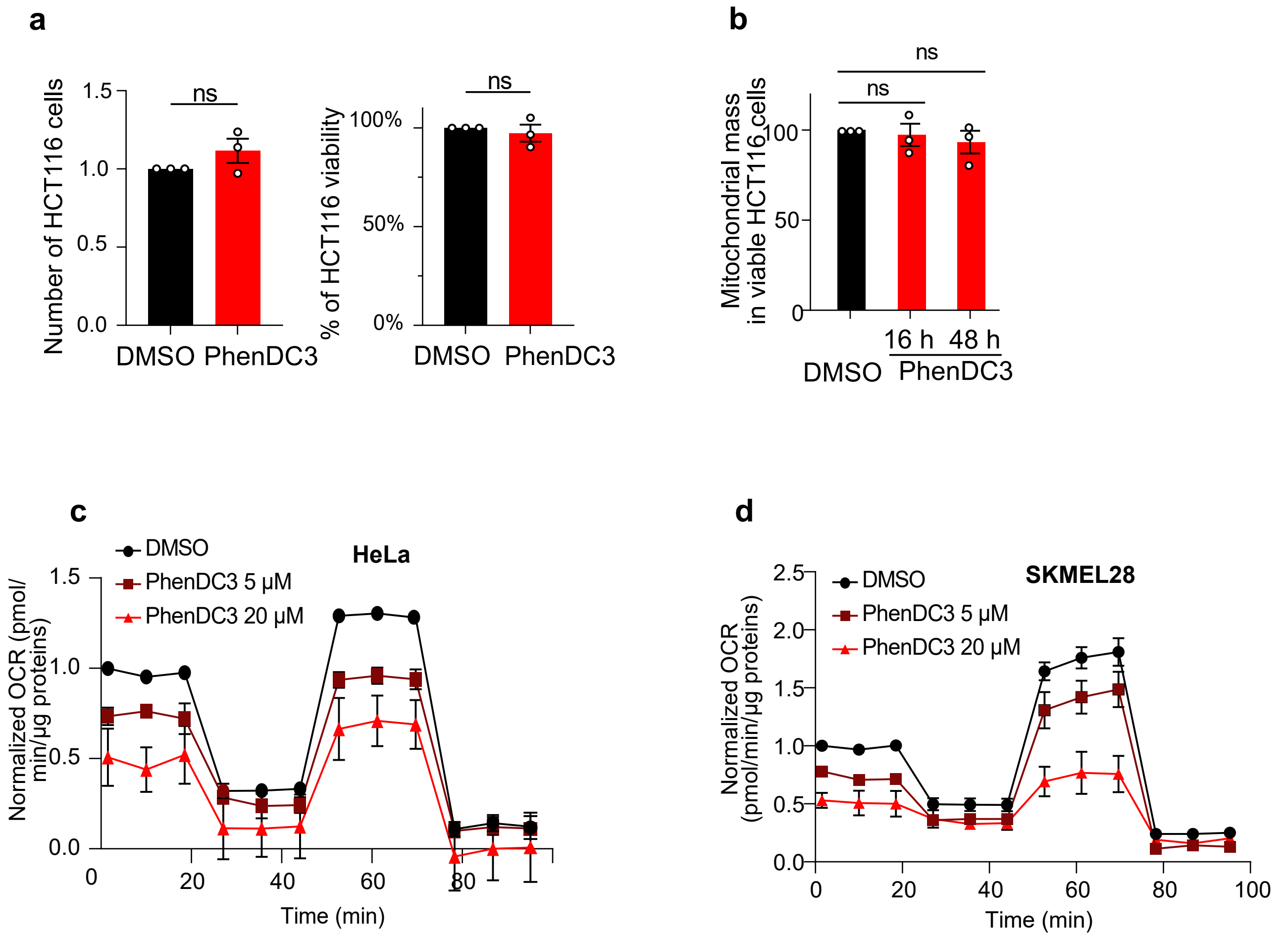

Supplementary Figure 2 (related to Figure 1). RG4 function in mitochondrial respiration. (a) HCT116 cell number and percentage of viability, followed by trypan blue cell counting, after treatments with DMSO and 20 μM of PhenDC3 for 24 h. Data are presented as mean values ± SEM of n=3 independent experiments, ns: non-significant (two-sided paired t-test). **(b)** Quantification of the mitochondrial mass followed by flow cytometry analysis of the fluorescent MTG probe of HCT116 cells treated with DMSO or 20 μM of PhenDC3 for 16 h and 48 h and plotted relatively to the DMSO condition. Data are presented as mean values ± SEM of n=3 independent experiments, ns: non-significant (two-sided paired t-test). **(c,d)** Quantification of the normalized Oxygen Consumption Rate (OCR) per cell, measured by a Seahorse assay, in HeLa (c) and SKMEL28 (d) cells treated with DMSO or a dose scale of PhenDC3 for 16 h. Data are presented as mean values ± SEM of n=3 independent experiments. Source data are provided as a Source Data file.

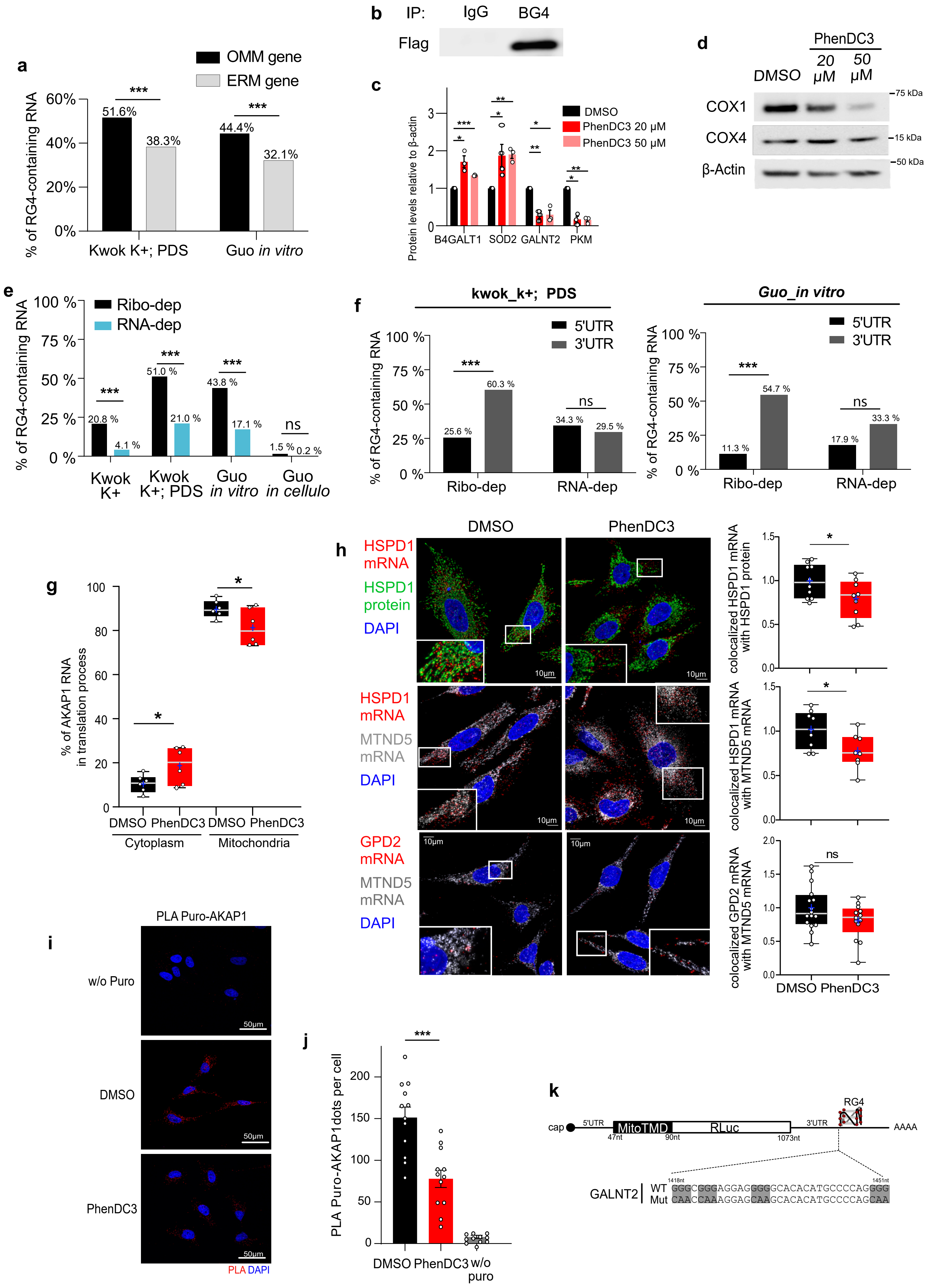
a
OMM gene
ERM gene
% of RG4-containing RNA
60%
40%
20%
0%
***
51.6%
38.3%
44.4%
32.1%
Kwok K+; PDS
Guo in vitro
b
IP:
IgG
BG4
Flag
c
Protein levels relative to β-actin
DMSO
PhenDC3 20 μM
PhenDC3 50 μM
B4GALT1
SOD2
GALNT2
PKM
d
PhenDC3
DMSO
20 μM
50 μM
COX1
COX4
β-Actin
75 kDa
15 kDa
50 kDa
e
% of RG4-containing RNA
Ribo-dep
RNA-dep
100 %
75 %
50 %
25 %
0 %
20.8 %
4.1 %
51.0 %
21.0 %
43.8 %
17.1 %
1.5 %
0.2 %
ns
Kwok K+
Kwok K+; PDS
Guo in vitro
Guo in cellulo
f
kwok_k+; PDS
Guo_in vitro
5'UTR
3'UTR
% of RG4-containing RNA
25.6 %
60.3 %
34.3 %
29.5 %
11.3 %
54.7 %
17.9 %
33.3 %
Ribo-dep
RNA-dep
g
% of AKAP1 RNA in translation process
100
80
60
40
20
0
DMSO
PhenDC3
Cytoplasm
Mitochondria
h
DMSO
PhenDC3
HSPD1 mRNA
HSPD1 protein
DAPI
MTND5 mRNA
GPD2 mRNA
10μm
colocalized HSPD1 mRNA with HSPD1 protein
colocalized HSPD1 mRNA with MTND5 mRNA
colocalized GPD2 mRNA with MTND5 mRNA
ns
i
PLA Puro-AKAP1
w/o Puro
DMSO
PhenDC3
50μm
PLA DAPI
j
PLA Puro-AKAP1 dots per cell
200
150
100
50
0
***
DMSO
PhenDC3
w/o puro
k
cap
5'UTR
MitoTMD
RLuc
3'UTR
RG4
AAAA
47nt
90nt
1073nt
1418nt
1451nt
GALNT2
WT GGGCGGGAGGAGGGGCACACATGCCCCAGGGG
Mut CAACCAAAGGAGCAAGCACACATGCCCCAGCAA

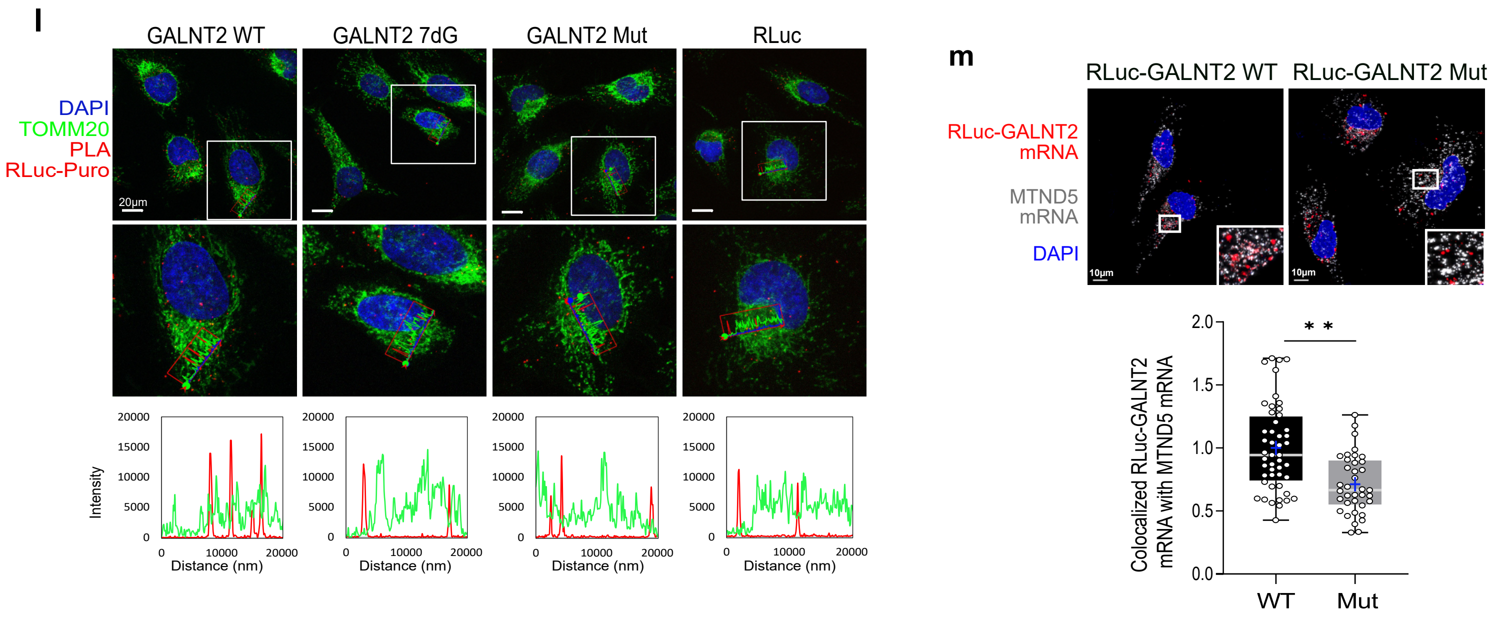

Supplementary Figure 3 (related to Figure 2). Analysis of RG4s in nuclear-encoded mitochondrial mRNAs (a) Proportion of RG4-containing mRNAs experimentally validated based on Kwok *et al* and Guo *et al* [1,2] localized at the OMM and the Endoplasmic Reticulum Membrane (ERM) based on Fazal *et al* [4]. For the RG4-containing mRNAs experimentally validated in K+ and PDS+ conditions based on Kwok *et al,* *P*=1.297e-09 and for the RG4-containing mRNAs experimentally validated *in vitro* based on Guo *et al*, *P*= 9.346e-09 (chi square test of proportions OMM vs ERM). **(b)** Immunoprecipitation of *in cellulo* RNA-protein complexes in HCT116 cells (cytoplasmic fraction) using the Flag-tagged BG4 antibody or control IgG, followed by western blot analysis of the Flag epitope tag. Shown is a representative result from n=3 independent experiments. **(c)** Quantification of the protein levels analyzed by western blot in Fig. 2b for B4GALT1, SOD2, GALNT2, PKM, normalized to β-actin and plotted relatively to DMSO condition. Data are presented as mean values ± SEM of n=3 independent experiments, *P<0.05, **P<0.01, ***P<0.001 (one-sided paired t-test). Data are provided as a Source Data file. **(d)** Western blot analysis of COX1, COX4 and β-Actin in HCT116 cells treated with dose scale of PhenDC3 for 48 h. The blot shown is a representative result from n=3 independent experiments. **(e)** Proportion of RG4-containing mRNAs experimentally validated based on Kwok *et al* and Guo *et* al [1,2] in ribosome (ribo)- and RNA-dependent nuclear-encoded mitochondrial mRNAs based on Fazal *et al* [4]. For the RG4-containing mRNAs experimentally validated in K+ and/or PDS+ conditions based on Kwok *et al* [2] or *in vitro* based on Guo *et al* , *P*<2.2e-16; for the RG4-containing mRNAs experimentally validated *in cellulo* based on Guo *et al* [1] *P*=0.07348 (chi square test of proportions ribo- vs RNA-dependent mRNAs). **(f)** Proportion of ribosome (ribo)- and RNA-dependent nuclear-encoded mitochondrial mRNAs based on Fazal *et al* [4] containing RG4 in 5'UTR and 3'UTR based on Kwok *et al* and Guo *et* al [1,2]. For the RG4-containing mRNAs experimentally validated in K+ and PDS+ conditions based on Kwok *et al*, *P*< 2.2e-16 and *P*= 0.5537 for ribo- and RNA-dependent mRNAs, respectively. For the RG4-containing mRNAs experimentally validated in vitro based on Guo *et al, P*< 2.2e-16 and *P*= 0.5537 for ribo- and RNA-dependent mRNAs, respectively (chi square test of proportions 5'UTR- vs 3'UTR).

Supplementary Figure 3 (related to Figure 2). Analysis of RG4s in nuclear-encoded mitochondrial mRNAs. (g) Distribution of AKAP1 mRNA translation sites, identified by the co-localization between AKAP1 mRNA (analyzed by RNAscope) and AKAP1 protein localization (analyzed by immunofluorescence (IF)), at the mitochondria or in the cytoplasm (co-localizing or not with MTND5 mitochondrial mRNA, respectively) of HeLa cells treated with DMSO or 20 µM PhenDC3 for 24 h. Data represent n=2 independent experiments and each dot is an image, **P*-value= 0.05, (two-sided paired t-test). Source data are provided as a Source Data file. **(h)** RNAscope analysis of HSPD1, GPD2 and MTND5 mRNA localization and IF analysis of HSPD1 protein localization in HeLa cells treated with DMSO, 20 µM PhenDC3 for 24 h. Scale bar indicates 10 µm. Shown is a single representative field from one experiment over n=2 or n=1 independent experiments for HSPD1 or GPD2, respectively. The graphs display the normalized HSPD1 protein fluorescence intensities around the HSPD1 mRNA or the co-localization between HSPD1 and MTND5 or GPD2 and MTND5 and were plotted relatively to the DMSO condition. Data represent n=2 or n=1 independent experiments for HSPD1 or GPD2, respectively and each dot is a cell, *P*-value= 0.0182 and *P*-value=0.011 for colocalized HSPD1 mRNA with HSPD1 protein and MTND5 mRNA, respectively, ns: non-significant (two-sided paired t-test). Source data are provided as a Source Data file. **(i)** Proximity Ligation Assay coupled with Puromycin Labeling (PLA-Puro) of AKAP1 in HeLa cells treated with DMSO or 20 µM PhenDC3 for 24 h. The specificity of the PLA signals was controlled with a condition without puromycin labelling (w/o Puro). Scale bar indicates 50 µm. Shown is a single representative field from one experiment over n=3 independent experiments. **(j)** Quantification of the number of dots per cell from the PLA Puro-AKAP1 images shown in Fig. 2h. Data are presented as mean values ± SEM of n=3 independent experiments and each dot is a cell, *P*-value= 0.0002 (two-sided paired t-test). The specificity of the PLA signal was controlled with a condition without puromycin labelling (w/o Puro). **(k)** Illustration of the capped and polyadenylated mRNA reporters containing the Mitochondrial Transmembrane Domain (MitoTMD) of AKAP1 fused to the Renilla Luciferase (RLuc) open reading frame and containing the 3'UTR of GALNT2. The 3'UTR region carry the experimentally identified RG4 forming sequence wild type (WT) or mutated (Mut), in which Gs are replaced by CAA in each stretch of guanines (in gray) forming the RG4. **(l)** PLA-Puro of RLuc-GALNT2 reporter mRNAs containing the RG4 unmodified (WT), mutated (Mut) or 7-deaza-modified (7dG) or RLuc reporter mRNA, combined with an IF analysis of TOMM20 protein in HeLa cells. Scale bar indicates 10 µm. Shown is a single representative field from one experiment over n=3 independent experiments. Graphs displaying the fluorescence intensity (arbitrary unit) in each channel over the distance depicted in the enlarged area. **(m)** RNAscope analysis and quantification of the localization of *in vitro* transcribed Rluc-GALNT2 reporter mRNA containing the RG4 WT or Mut and MTND5 mRNA in HeLa cells. Scale bar indicates 10 µm. Shown is a single representative field from one experiment over n=3 independent experiments. Data are presented as mean values ± SEM of n=3 independent experiments and each dot is a cell, *P*-value= 0.0011 (two-sided paired t-test). Source data are provided as a Source Data file. For (g), (h), (m) the box extends from the 25th to 75th percentiles; the line in the middle of the box is plotted at the median and the "+" at the mean; the whiskers show the upper and lower extremes.

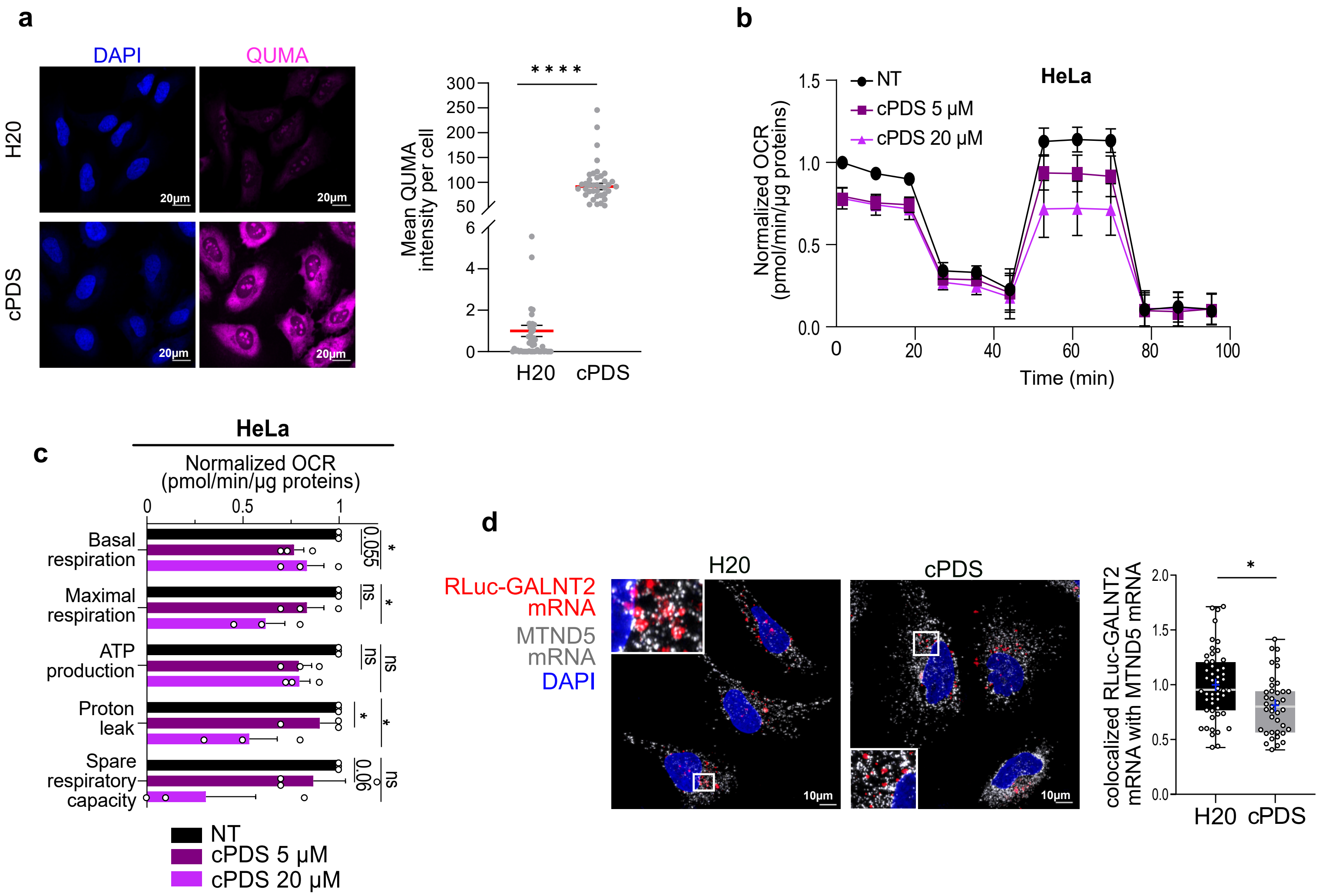

Supplementary Figure 4 (related to Figure 2). Effect of cPDS on RG4 formation, mitochondrial respiration and RG4-mRNA localization at mitochondria (a) RG4 detection in fixed cells using the QUMA probe (1 µM for 24 h) in HeLa cells treated with 20 µM of cPDS for 24 h. Scale bar indicates 20 µm. The graphs display the quantification of the QUMA signal intensity per cells plotted relatively to the control condition (H20). Number of cells counted : 49 cells for H20, 43 cells for cPDS. Data are presented as mean values ± SEM and each dot is a cell, *P*-value<0.0001 (two-sided paired t-test). **(b)** Quantification of the normalized Oxygen Consumption Rate (OCR) per cell, measured by a Seahorse assay in HeLa cells treated with a dose scale carboxypyridostatin (cPDS) for 16 h. Data are presented as mean values ± SEM of n=3 independent experiments. **(c)** Quantification of the Oxygen Consumption Rate (OCR) linked to basal and maximal mitochondrial respiration, ATP turnover, proton leak, and spare respiratory capacity, measured by Seahorse assay, in HeLa cells treated with a dose scale of cPDS for 16 h and plotted relatively to the untreated condition (NT). Data are presented as mean values ± SEM of n=3 independent experiments, *P<0.05, ns: non-significant (two-sided paired t-test). **(d)** RNAscope analysis of Rluc-GALNT2 reporter mRNA (capped and polyadenylated depicted in Supplementary Fig. 3g) and MTND5 mRNA localization in HeLa cells treated with H20 or 20 µM carboxypyridostatin (cPDS) for 24 h. Scale bar indicates 10 µm. Shown is a single representative field from one experiment over n=3 independent experiments. Quantification of the co-localization between RLuc-GALNT2 mRNA reporter and MTND5 mRNA. The box extends from the 25th to 75th percentiles; the line in the middle of the box is plotted at the median and the "+" at the mean; the whiskers show the upper and lower extremes. Data are presented as mean values ± SEM of n=3 independent experiments and each dot is a cell, *P*-value= 0.0246 (two-sided paired t-test). For all the panels, source data are provided as a Source Data file.

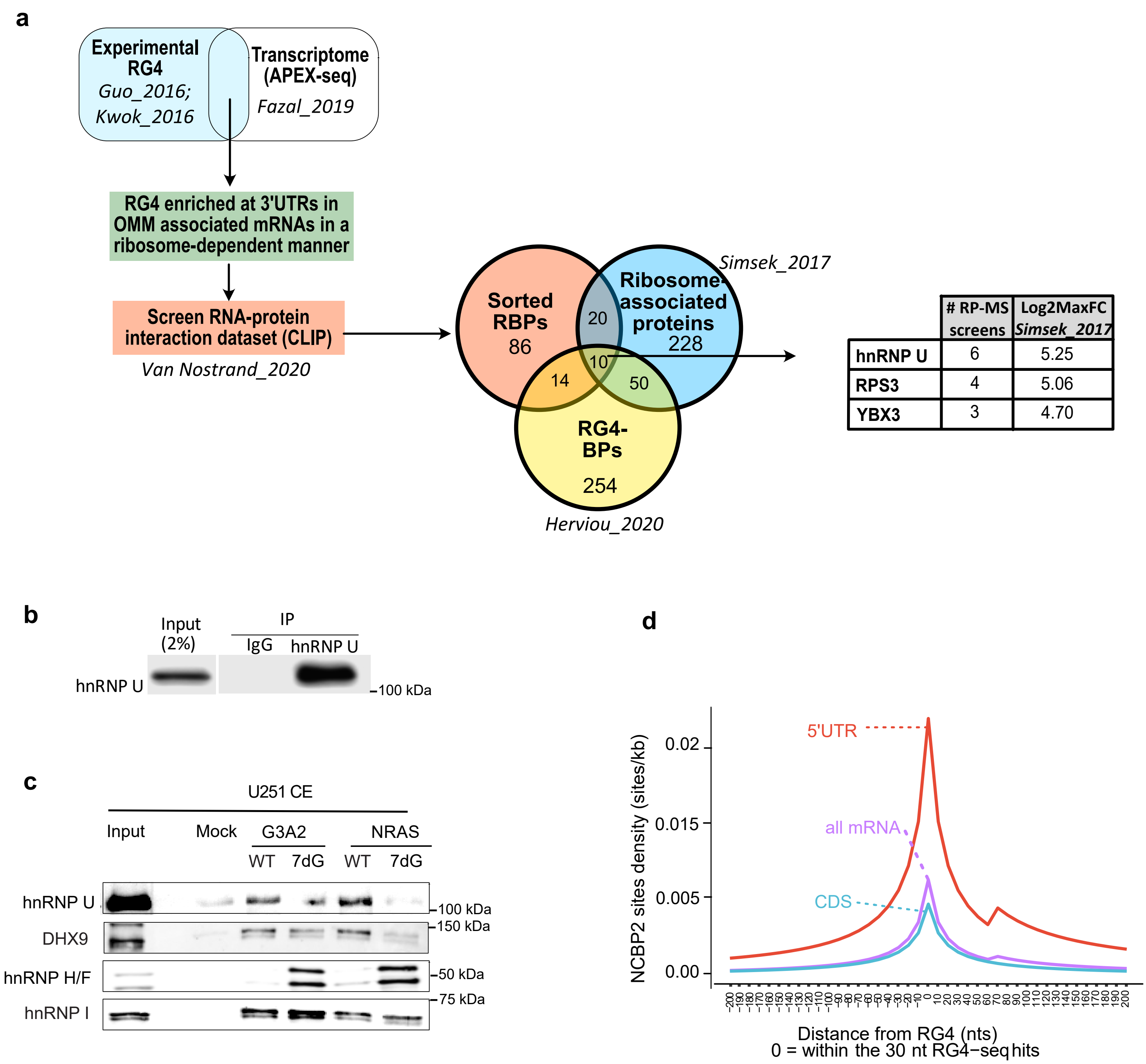

Supplementary Figure 5 (related to Figure 3). Identification of hnRNP U as a regulator of RG4-containing nuclear-encoded mitochondrial mRNAs. (a) Overview of filtering parameters to identify RBPs bound to RG4s located in the 3'UTRs of OMM-associated mRNAs in a ribosome-dependent manner mRNAs. **(b)** Immunoprecipitation (IP) of *in cellulo* RNA-protein complexes in cytoplasmic extracts from HCT116 cells with the hnRNP U antibody. **(c)** RNA affinity chromatography using the G3A2 and NRAS RG4 sequences either native (WT) or 7-deaza-modified (7dG) and U251 cytoplasmic cell extracts, followed by western blot analysis of hnRNP U, DHX9, hnRNP H/F and hnRNP I. Shown is a representative result from n=2 independent experiments for G3A2 sequences, n=1 for NRAS RG4 sequences. Source data are provided as a Source Data file. **(d)** Density of NCBP2 binding sites around RG4s (based on [2]) located in mRNA (all mRNA), CDS, and 5'UTR. There are no such binding sites for the 3'UTR region and the corresponding line was therefore not drawn. At distance 0: P = ns (non-significant).

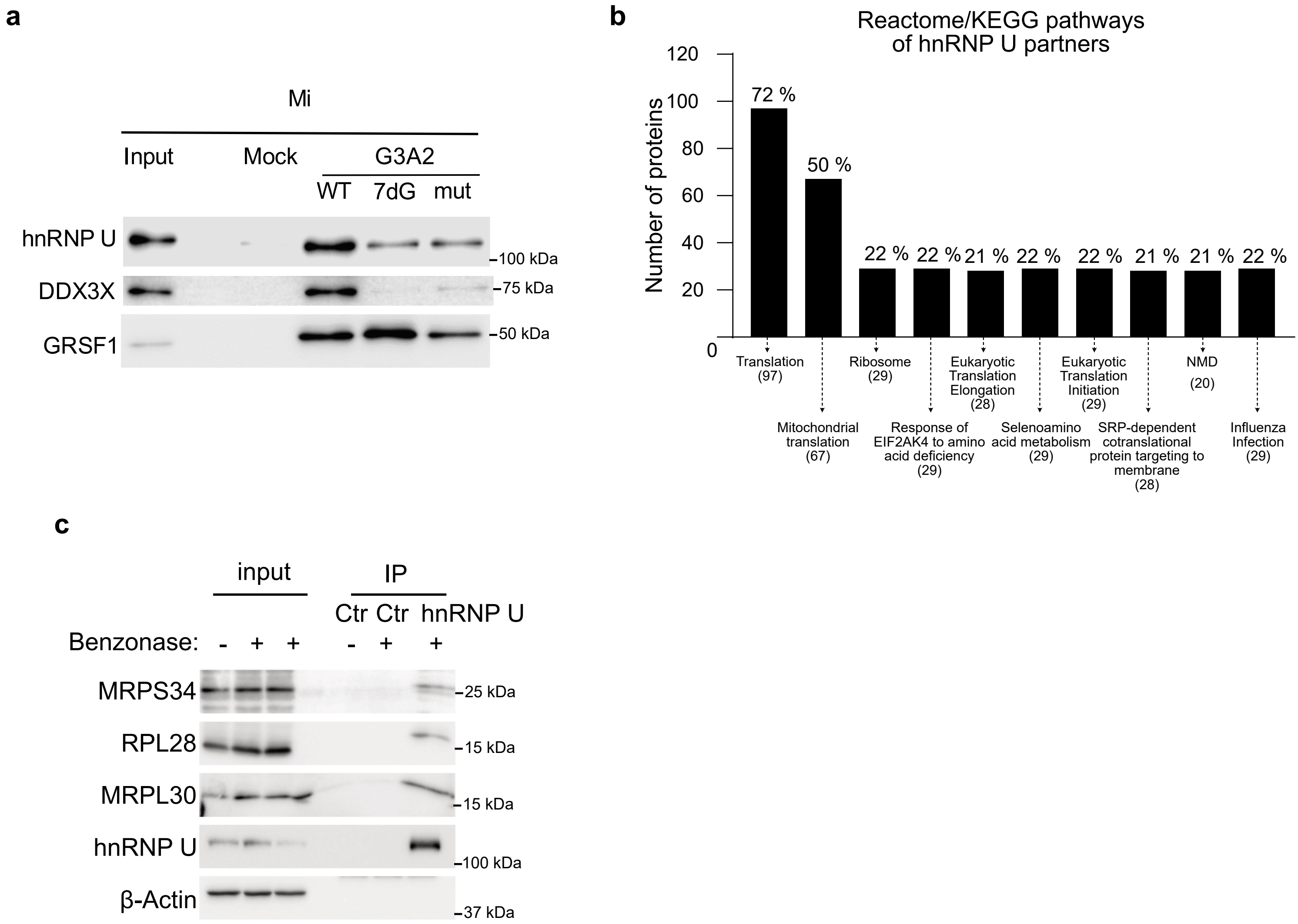

Supplementary Figure 6 (related to Figure 4). hnRNP U binding to RG4s in mitochondrial fractions (a) RNA affinity chromatography using the G3A2 and NRAS RG4 sequences either native (WT), 7-deaza-modified (7dG) or mutated (mut) and HCT116 mitochondrial cell extracts (Mi), followed by western blot analysis of hnRNP U, DDX3X and GRSF1. The blot shown is a representative result from n=2 independent experiments. Source data are provided as a Source Data file. **(b)** Functional enrichment analysis of the identified high confidence 134 hnRNP U partners. **(c)** Immunoprecipitation (IP) of HCT116 cytoplasmic extracts treated or not with benzonase using the hnRNP U antibody or control IgG, followed by western blot analysis and probing with the indicated antibodies. Shown is a representative result from n=3 independent experiments. Source data are provided as a Source Data file.

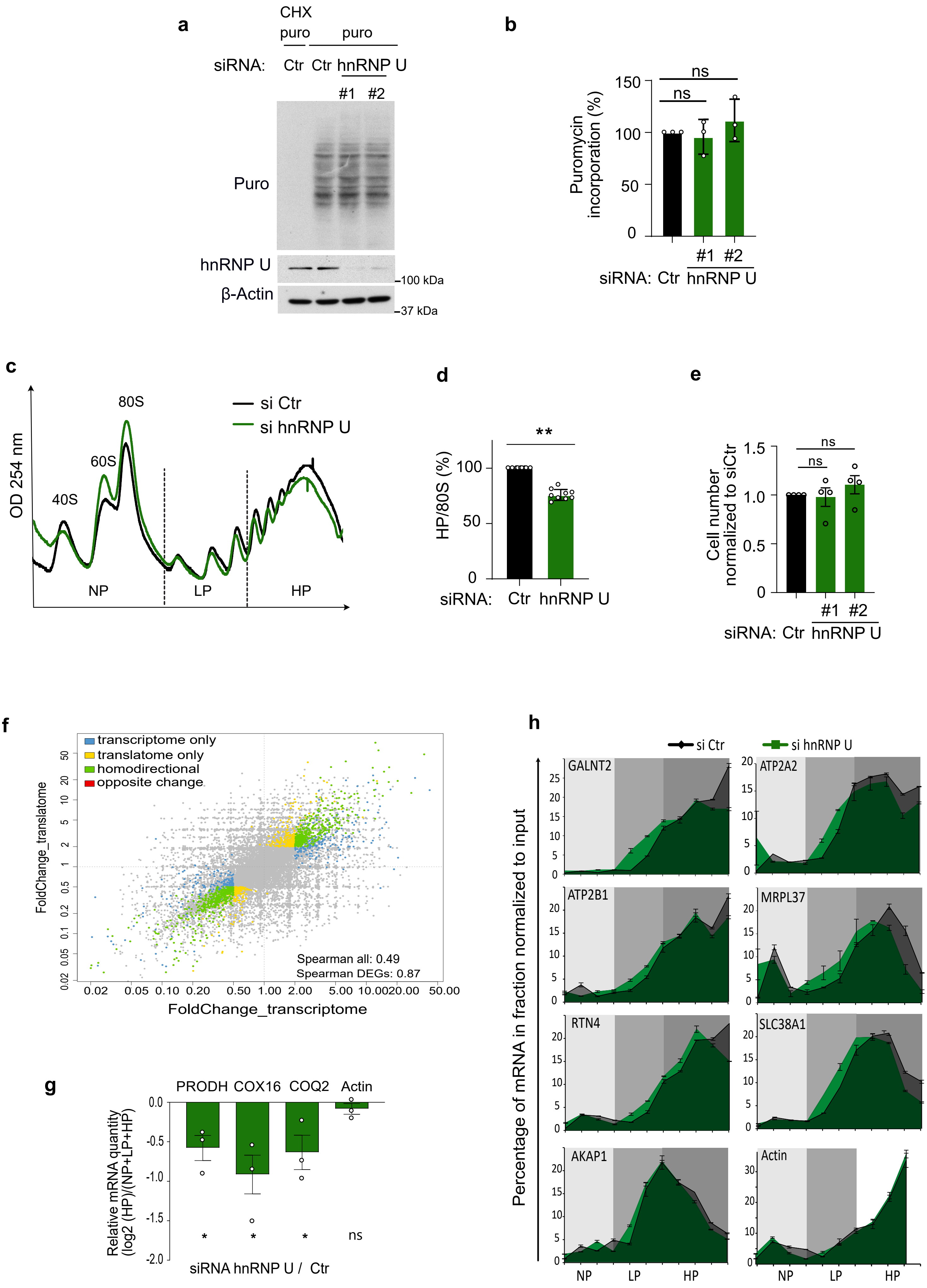

a
CHX puro
puro
siRNA: Ctr Ctr hnRNP U
#1 #2
Puro
hnRNP U
β-Actin
100 kDa
37 kDa
b
Puromycin incorporation (%)
ns
ns
siRNA: Ctr hnRNP U
#1 #2
c
OD 254 nm
40S
60S
80S
si Ctr
si hnRNP U
NP
LP
HP
d
HP/80S (%)
**
siRNA: Ctr hnRNP U
e
Cell number normalized to siCtr
ns
ns
siRNA: Ctr hnRNP U
#1 #2
f
transcriptome only
translatome only
homodirectional
opposite change
FoldChange_translatome
FoldChange_transcriptome
Spearman all: 0.49
Spearman DEGs: 0.87
g
PRODH COX16 COQ2 Actin
Relative mRNA quantity (log2 (HP)/(NP+LP+HP))
siRNA hnRNP U / Ctr
ns
h
si Ctr
si hnRNP U
Percentage of mRNA in fraction normalized to input
GALNT2
ATP2A2
ATP2B1
MRPL37
RTN4
SLC38A1
AKAP1
Actin
NP LP HP

Supplementary Figure 7 (related to Figure 5). Role of hnRNP U in translational regulation. **(a)** *De novo* protein synthesis analysis by SUnSET assay in HCT116 cells treated with control (Ctr), and 2 different hnRNP U (#1, #2) siRNAs, followed by western blot analysis of the incorporated puromycin, hnRNP U and β-Actin. The blot shown is a representative result from n=3 independent experiments. Source data are provided as a Source Data file. **(b)** Quantification of puromycin incorporation from (a) plotted relatively to the siRNA Ctr condition. Data are presented as mean values ± SEM of n=3 independent experiments, ns: non-significant (two-sided paired t-test). **(c)** Polysome profile of HCT116 cells treated with control (si Ctr) and hnRNP U (si hnRNP U) siRNAs. The positions of the 40S, 60S and 80S ribosomal subunits and non-polysomal (NP) and light (LP) and heavy (HP) polysomal fractions are indicated. Shown is a representative result from n=9 independent experiments. **(d)** Quantification of the ratio between the area under the HP curve and the 80S area from the polysome profile in (c) in the si Ctr and si hnRNP U conditions and plotted relatively to the si Ctr condition. Data are presented as mean values ± SEM of n=9 independent experiments, *P*-value= 4,61e-07 (two-sided paired t-test). **(e)** HCT116 cell number after treatments with si Ctr and si hnRNP U#1, si hnRNP U#2 plotted relatively to the si Ctr condition. Data are presented as mean values ± SEM of n=4 independent experiments, ns: non-significant (two-sided paired t-test). **(f)** Transcriptional and translational analysis of polysome associated mRNAs extracted after polysome profiling of (c). Data are presented as fold change in transcriptome and translatome between HCT116 cells treated with Ctr and hnRNP U siRNAs. Colour-coded are genes with the adjusted P-value < 0.05 with log2(fold change) >=1 (DESeq2); n=3 independent experiment per group. **(g,h)** RT-qPCR analysis from pooled (g) or individual (h) NP, LP and HP polysomal fractions extracted from the polysome profile in (c) using specific primers for the indicated mRNAs. Quantification by analyzing the ratio HP/total mRNAs in (g). Data are presented as mean values ± SEM of duplicates of n=3 independent experiments analyzed in (g) and of a representative experiment from n=2 independent experiments in (h). Source data and exact *P*-values are provided as a Source Data file.

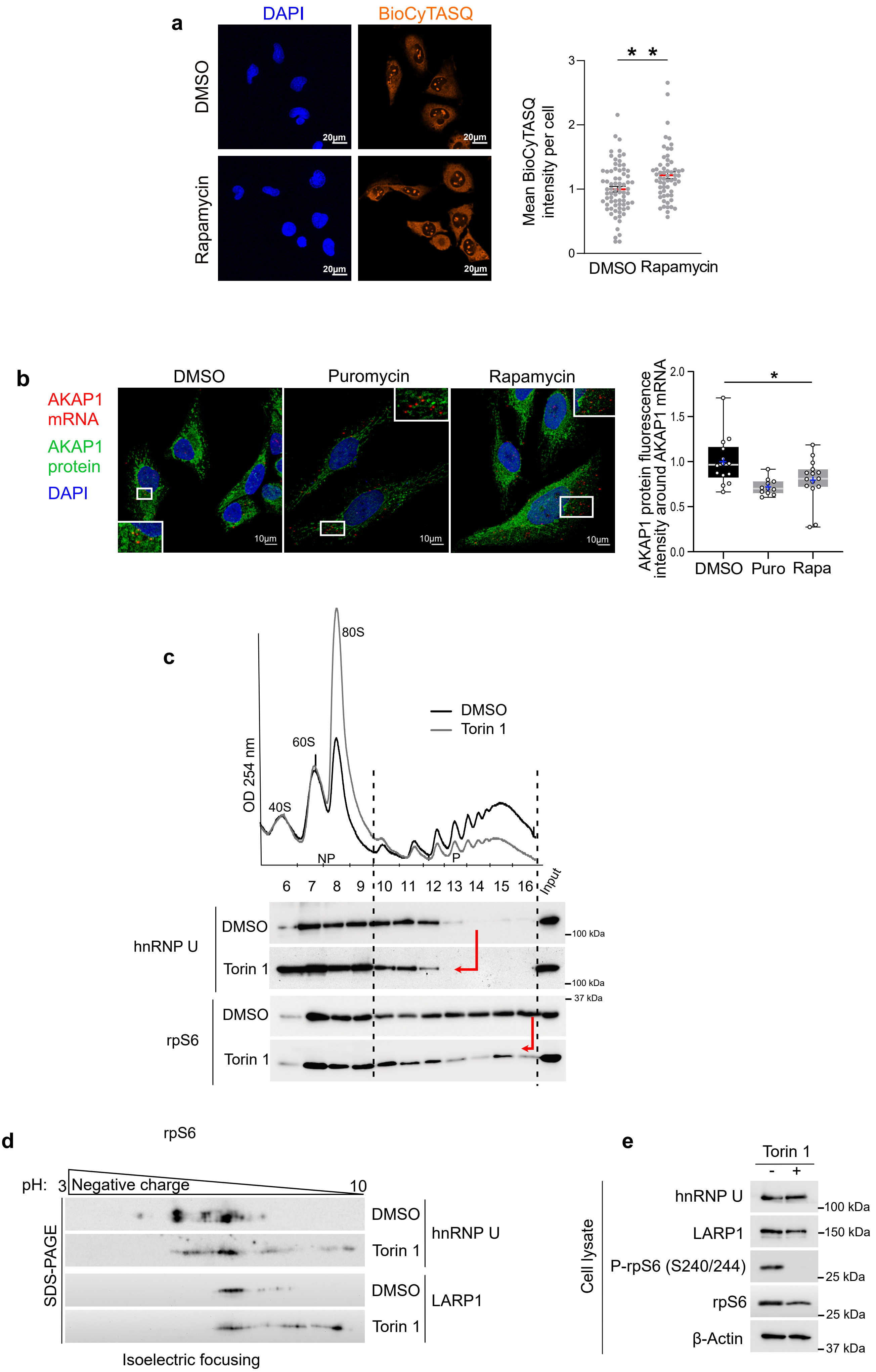
a
DAPI
BioCyTASQ
DMSO
Rapamycin
20µm
Mean BioCyTASQ intensity per cell
* *
DMSO Rapamycin
b
AKAP1 mRNA
AKAP1 protein
DAPI
DMSO
Puromycin
Rapamycin
10µm
AKAP1 protein fluorescence intensity around AKAP1 mRNA
*
DMSO Puro Rapa
c
80S
60S
40S
DMSO
Torin 1
OD 254 nm
NP
P
6 7 8 9 10 11 12 13 14 15 16 Input
hnRNP U
DMSO
Torin 1
100 kDa
37 kDa
rpS6
d
rpS6
pH: 3 Negative charge 10
SDS-PAGE
DMSO
Torin 1
hnRNP U
LARP1
Isoelectric focusing
e
Torin 1
- +
Cell lysate
hnRNP U
LARP1
P-rpS6 (S240/244)
rpS6
β-Actin
100 kDa
150 kDa
25 kDa
37 kDa

Supplementary Figure 8 (related to Figure 5). Role of mTOR in hnRNP U-mediated regulation of nuclear-encoded mitochondrial mRNA translation. (a) RG4 detection fixed cells using the BioCyTASQ probe (1 µM for 24 h) coupled with an immunofluorescence (IF) analysis of TOMM20 protein localization in HeLa cells treated with DMSO or 100 nM of Rapamycin for 2 h. Scale bar indicates 20 µm. The graph displays the quantification of the BioCyTASQ signal intensity plotted relatively to the DMSO condition. Data are presented as mean values ± SEM and each dot is a cell, *P*-value= 0.0012 (two-sided paired t-test). Number of cells counted: 74 cells for DMSO, 59 cells for Rapamycin. Source data are provided as a Source Data file. **(b)** RNAscope analysis of AKAP1 mRNA localization and IF analysis of AKAP1 protein localization in HeLa cells treated with DMSO or 100 nM of Rapamycin for 2 h and 100 µg/mL puromycin for 1 h. Scale bar indicates 10 µm. Shown is a single representative field from one experiment over n=3 independent experiments. The graphs display the normalized AKAP1 protein fluorescence intensities around the AKAP1 mRNA and were plotted relatively to the DMSO condition. The box extends from the 25th to 75th percentiles; the line in the middle of the box is plotted at the median and the "+" at the mean; the whiskers show the upper and lower extremes. Each dot is a cell, *P*-value= 0.035 (two-sided paired t-test). Number of cells counted: 14 cells for DMSO, 11 cells for Puromycin and 15 cells for Rapamycin. Source data are provided as a Source Data file. **(c)** Polysome profile of HCT116 cells treated with DMSO or 300 nM Torin 1 for 3 h, followed by western blot analysis from individual non-polysomal (NP) and polysomal (P) fractions by probing for hnRNP U and rpS6 (positive control). Shown is a representative result from n=3 independent experiments. **(d)** Isoelectric focusing followed by western blot analysis of hnRNP U and LARP1 in HCT116 cells treated with DMSO and 300 nM Torin 1 for 3 h. The blot shown is a representative result from n=2 independent experiments. **(e)** Western blot analysis of hnRNP U, LARP1, P-rpS6 (positive control), rpS6 and β-Actin in HCT116 cells treated with DMSO and 300 nM Torin 1 for 3 h. The blot shown is a representative result from n=2 independent experiments.

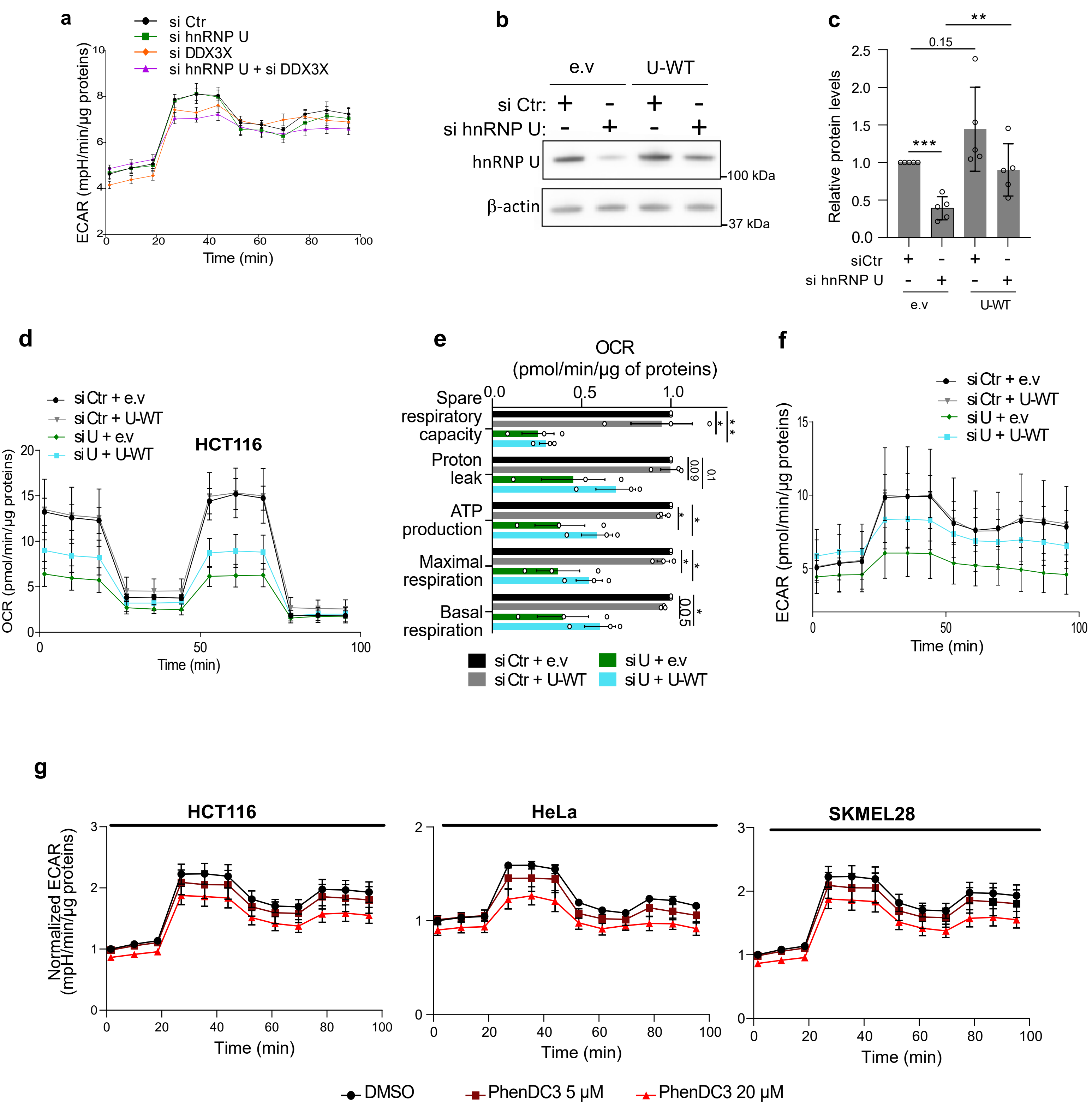

Supplementary Figure 9 (related to Figure 6). Role of hnRNP U in energy metabolism. (a) Quantification of the Extracellular Acidification Rate (ECAR) measured by a Seahorse assay (mitochondrial stress test), in HCT116 cells treated with control (si Ctr), hnRNP U (si hnRNP U), DDX3X (si DDX3X) or a combination of hnRNP U and DDX3X (si hnRNP U + si DDX3X) siRNAs for 48 h. Data are presented as mean values ± SEM of n=3 independent experiments. **(b)** Western blot analysis of hnRNP U and β-Actin in HCT116 cells treated with si Ctr or si hnRNP U for 72 h and transfected with an empty vector (e.v) or a plasmid expressing hnRNP U WT -FLAG for 48 h. The blot shown is a representative result from n=3 independent experiments.

(c) Quantification of the protein levels in (b) normalized to β-Actin and plotted relatively to si Ctr condition. Data are presented as mean values ± SEM of n=3 independent experiments, *P<0.05, **P<0.01, ***P<0.001, ns: non-significant (two-sided paired t-test). **(d)** Quantification of the normalized Oxygen Consumption Rate (OCR) per cell, measured by a Seahorse assay in HCT116 cells treated as in (b). Data are presented as mean values ± SEM of n=3 independent experiments. **(e)** Quantification of the Oxygen Consumption Rate (OCR) linked to basal and maximal mitochondrial respiration, ATP turnover, proton leak, and spare respiratory capacity, measured by Seahorse assay, in HCT116 cells treated as in (b). Data are presented as mean values ± SEM of n=3 independent experiments, *P<0.05, ns: non-significant (two-sided paired t-test). **(f)** Quantification of the Extracellular Acidification Rate (ECAR) measured by a Seahorse assay in HCT116 treated as in (b). **(g)** Quantification of the Extracellular Acidification Rate (ECAR) measured by a Seahorse assay, in HCT116, HeLa and SKMEL28 cells treated with with DMSO or a dose scale of PhenDC3 for 16 h. Data are presented as mean values ± SEM of n=3 independent experiments. For all the panels, Source data and exact *P*-values are provided as a Source Data file.

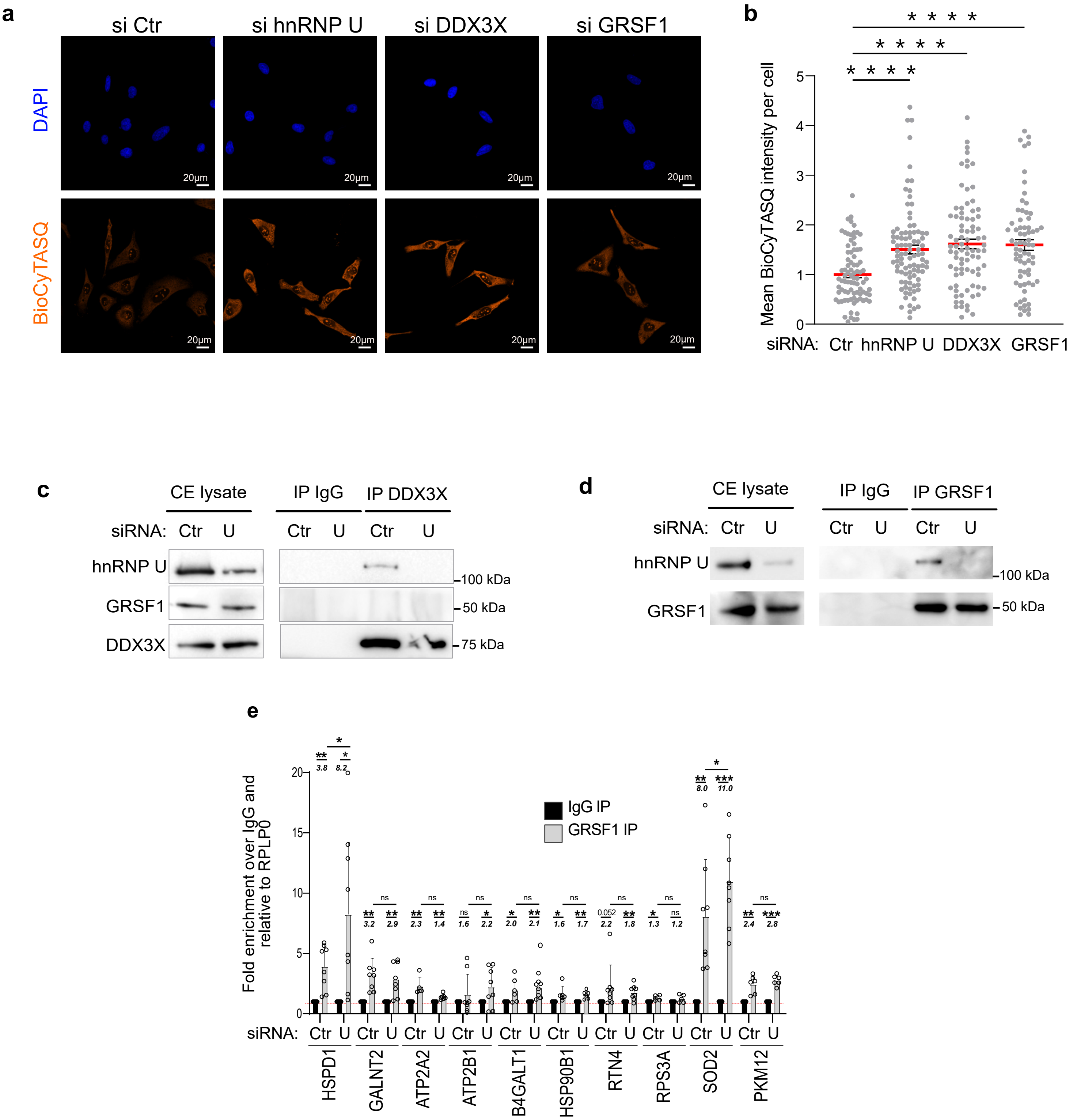

Supplementary Figure 10 (related to Figure 7). Collaboration between hnRNP U, DDX3X and GRSF1 to regulate RG4-dependent translation. (a) RG4 detection using the BioCyTASQ of HeLa cells treated with control (si Ctr), hnRNP U (si hnRNP U), DDX3X (si DDX3X), GRSF1 (si GRSF1) siRNAs for 48 h. Scale bar indicates 20 µm. Shown is a representative result from n=3 independent experiments. **(b)** Quantification of the BioCyTASQ signal intensity from (a) plotted relatively to the siCtr condition. Data are presented as mean values ± SEM of n=3 independent experiments and each dot is a cell, ****P<0.0001 (two-sided unpaired t-test). Source data are provided as a Source Data file. **(c)** Immunoprecipitation (IP) with the DDX3X antibody of *in cellulo* RNA-protein complexes in cytoplasmic extracts (CE) from HCT116 cells treated with the si Ctr or si hnRNPU followed by western blot analysis and probing with the indicated antibodies.

(d) IP of *in cellulo* RNA-protein complexes in CE from HCT116 cells with the GRSF1 antibody. **(e)** RT-qPCR analysis of the indicated mRNAs present in the *in cellulo* RNA-protein complexes from (d). The relative mRNA levels for each IP sample were normalized to the RPLP0 mRNA (negative control) and to the corresponding IP IgG. The italic numbers indicated above each histograms correspond to the fold increase of the mRNA levels in the IP relatively to the IgG. Data are presented as mean values ± SEM of n=3 independent experiments each consisting of duplicates, *P<0.05, **P<0.01, ***P<0.001, ns: non-significant (one-sided paired t-test). Source data and exact *P*-values are provided as a Source Data file.

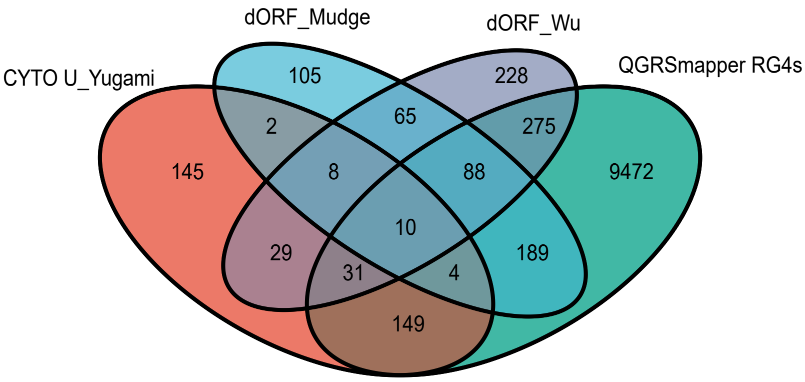

Supplementary Figure 11. Molecular mechanism underlying translational regulation by 3'UTR RG4s. Venn diagram showing the overlap of mRNAs bound by hnRNP U in the cytoplasm from Yugami *et al*. [5] with the mRNAs containing dORF identified in Wu *et al.*[6] and Mudge *et al.*[7], and mRNAs containing RG4s predicted by QGRSmapper[8].

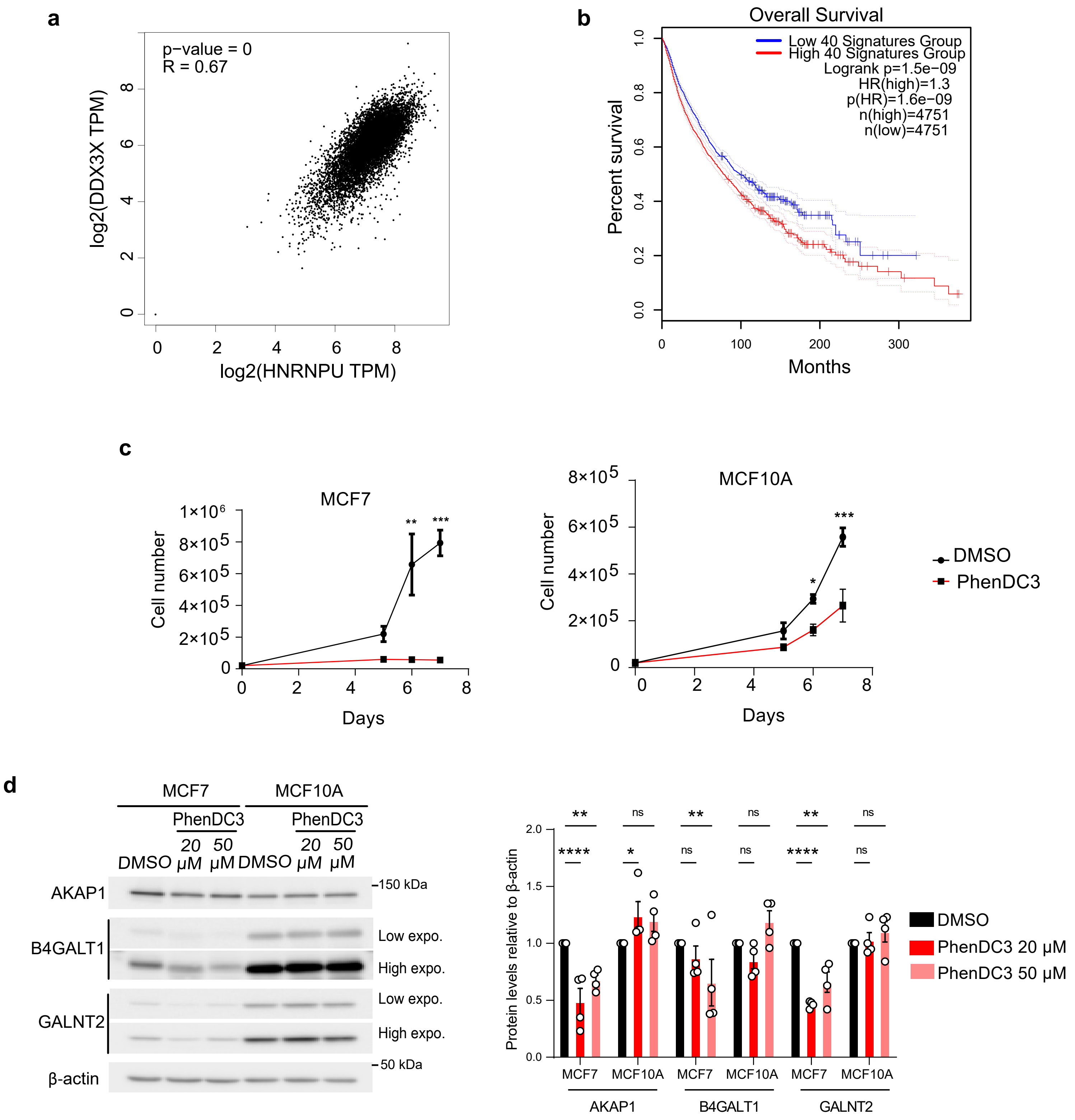

Supplementary Figure 12. hnRNP U expression and prognostic value in cancer (a) Expression correlation analysis of hnRNP U and DDX3X using the GEPIA2 database[84]. The Spearman correlation coefficient was used. **(b)** Representation of Kaplan-Meier Overall Survival curve using the GEPIA2 database[84]. **(c)** Proliferation in MCF7 and MCF10A cells treated with DMSO or 20 μM PhenDC3 for the indicated time. Data are presented as mean values ± SEM of n=3 independent experiments. For all the panels, *P<0.05, **P<0.01, ***P<0.001, ns: non-significant (two-way ANOVA). Source data and exact *P*-values are provided as a Source Data file. **(d)** Western blot analysis and quantification of AKAP1, GALNT2, B4GALT1 and β–Actin in MCF7 or MCF10A cells treated with DMSO, 20 or 50 μM PhenDC3 for 48 h. Shown is a representative blot of n=4 independent experiments. Source data and exact *P*-values are provided as a Source Data file.

SUPPLEMENTARY REFERENCES

1 Guo, J. U. & Bartel, D. P. RNA G-quadruplexes are globally unfolded in eukaryotic cells and depleted in bacteria. *Science* **353**, doi:10.1126/science.aaf5371 (2016).

2 Kwok, C. K., Marsico, G., Sahakyan, A. B., Chambers, V. S. & Balasubramanian, S. rG4-seq reveals widespread formation of G-quadruplex structures in the human transcriptome. *Nature methods* **13**, 841-844, doi:10.1038/nmeth.3965 (2016).

3 Herviou, P. *et al.* hnRNP H/F drive RNA G-quadruplex-mediated translation linked to genomic instability and therapy resistance in glioblastoma. *Nature communications* **11**, 2661, doi:10.1038/s41467-020-16168-x (2020).

4 Fazal, F. M. *et al.* Atlas of Subcellular RNA Localization Revealed by APEX-Seq. *Cell* **178**, 473-490 e426, doi:10.1016/j.cell.2019.05.027 (2019).

5 Yugami, M., Okano, H., Nakanishi, A. & Yano, M. Analysis of the nucleocytoplasmic shuttling RNA-binding protein HNRNPU using optimized HITS-CLIP method. *PloS one* **15**, e0231450, doi:10.1371/journal.pone.0231450 (2020).

6 Wu, Q. *et al.* Translation of small downstream ORFs enhances translation of canonical main open reading frames. *The EMBO journal* **39**, e104763, doi:10.15252/embj.2020104763 (2020).

7 Mudge, J. M. *et al.* Standardized annotation of translated open reading frames. *Nat Biotechnol* **40**, 994-999, doi:10.1038/s41587-022-01369-0 (2022).

8 Bourdon, S. *et al.* QUADRatlas: the RNA G-quadruplex and RG4-binding proteins database. *Nucleic acids research*, doi:10.1093/nar/gkac782 (2022).
