# Supplementary material for: RNA G-quadruplexes control mitochondria-localized mRNA translation and energy metabolism
Source: Nat Commun. 2025 Apr 7;16:3292. doi: 10.1038/s41467-025-58118-5 (PMC11977240; doi:10.1038/s41467-025-58118-5)
Supplement: Supplementary file 2 — Description of Additional Supplementary Files [file 41467_2025_58118_MOESM2_ESM.pdf]

Description of additional supplementary files:

File Name: Supplementary Data 1
Description: IP-MS analysis of hnRNP U interactors

File Name: Supplementary Data 2
Description: List of primers used in RT-qPCR and for cloning

File Name: Supplementary Data 3
Description: List of primers for generating the DNA templates used for the synthesis of biotinylated RNAs.
